# Effect of Possible Osteoporosis on Parenchymal-Type Hemorrhagic Transformation in Patients with Cardioembolic Stroke

**DOI:** 10.3390/jcm10112526

**Published:** 2021-06-07

**Authors:** Yu-Deok Won, Jae-Min Kim, Jin-Hwan Cheong, Je-Il Ryu, Seong-Ho Koh, Myung-Hoon Han

**Affiliations:** 1 Department of Neurosurgery, Hanyang University Guri Hospital, 153 Gyeongchun-ro, Guri 471-701, Gyonggi-do, Korea; hidma823@hanmail.net (Y.-D.W.); kjm2323@hanyang.ac.kr (J.-M.K.); cjh2324@hanyang.ac.kr (J.-H.C.); ryujeil@hanyang.ac.kr (J.-I.R.); 2 Department of Neurology, Hanyang University Guri Hospital, 153 Gyeongchun-ro, Guri 471-701, Gyonggi-do, Korea; ksh213@hanyang.ac.kr

**Keywords:** hemorrhagic transformation, cardioembolic stroke, parenchymal type hematoma, bone mineral density, osteoporosis, Hounsfield unit

## Abstract

Background: hemorrhagic transformation (HT) is a frequent complication of ischemic stroke, and parenchymal hematoma (PH)-type HT has been shown to correlate with symptomatic deterioration. Because both bone and vascular smooth muscle cells are composed of type 1 collagen, we hypothesized that the integrity of blood vessels around the infarction area might be more damaged in osteoporotic conditions after a cardioembolic stroke. Methods: we measured frontal skull Hounsfield unit (HU) values on brain CT images from cardioembolic stroke patients. We conducted a receiver operating characteristic curve analysis in a large sample registry to identify the optimal HU threshold for predicting osteopenia and osteoporosis. Hazard ratios were estimated using a Cox regression analysis to identify whether osteoporotic conditions were an independent predictor of PH-type HT in patients with cardioembolic stroke. Results: altogether, 600 consecutive patients (>18 years old) with cardioembolic stroke were enrolled over a 12-year period at our hospital. The infarction volume and hypothetical osteoporosis were independent predictive factors for PH-type HT development in patients with cardioembolic stroke. In the male group, hypothetical osteoporosis was an independent predictor for PH-type HT development after cardioembolic stroke (hazard ratio, 4.12; 95% confidence interval, 1.40–12.10; *p* = 0.010). Conclusions: our study suggests an association between possible osteoporosis and the development of PH-type HT in patients with cardioembolic stroke. Our findings could help to predict PH-type HT by providing a convenient method for measuring the HU value using brain CT images.

## 1. Introduction

Hemorrhagic transformation (HT) is a frequent complication of acute ischemic stroke. The incidence of spontaneous HT ranges from 38% to 71% in autopsy studies and from 13% to 43% in CT studies, whereas the incidence of symptomatic HT ranges from 0.6% to 20% [1]. HT is divided into hemorrhagic infarction (HI) and parenchymal hematoma (PH) types [2]. Patients with HI-type HT have no significantly higher risk of neurological deterioration than patients without HT, but patients with PH-type HT have symptomatic clinical deterioration [1,2,3,4]. According to a previous study, only PH-type HT in ischemic stroke is associated with early or late symptomatic neurological deterioration; we therefore wanted to focus on the risk factors associated with the occurrence of PH-type HT [4]. Cardioembolic stroke is more associated with a high risk of symptomatic HT than other types of ischemic stroke [1,5]. Therefore, we included only cardioembolic stroke patients in this analysis to reduce the effects of heterogeneity among the subtypes of ischemic stroke on our results.

Both bone and vascular smooth muscle cells are composed of type 1 collagen. Osteoporosis is a systemic disease that is strongly associated with genetic components of type 1 collagen [6]. Therefore, we previously hypothesized that low bone mineral density (BMD) would negatively influence the integrity of bridging veins, as veins are composed of smooth muscle cells [7]. Loss and degenerative changes in vascular smooth muscle cells negatively influence vascular strength, which can cause blood vessels to rupture easily [8,9].

Therefore, in this study, we hypothesized that after a cardioembolic stroke, the integrity of blood vessels around the infarction area might be more damaged in patients with osteoporotic conditions than in other patients. We also conjecture that that damage might be associated with a higher prevalence of PH-type HT in osteoporotic patients. To assess that hypothesis, we measured Hounsfield unit (HU) values in the frontal skulls of patients with cardioembolic stroke and examined the association between hypothetical BMD and PH-type HT by using the HU threshold for predicting osteopenia and osteoporosis from a large sample registry.

## 2. Materials and Methods

### 2.1. Study Patients

We retrospectively extracted data for all consecutive patients with acute ischemic stroke, from 1 January 2009 to 31 December 2020, from the registry of ischemic stroke patients at Hanyang University Guri Hospital, Korea. In the registry, all ischemic stroke subtypes are classified according to the TOAST classification [10]. As explained in the Introduction, we included only cardioembolic stroke patients in this study. We initially identified 703 suitable patients. All patients were confirmed to have had a cardioembolic stroke through brain magnetic resonance imaging (MRI), including diffusion-weighted imaging; electrocardiogram; laboratory studies; carotid Doppler imaging; echocardiography. Because brain computed tomography (CT) was needed to measure the frontal skull HU value, we excluded 62 patients who did not have at least one brain CT image around the time of their cardioembolic stroke diagnosis. To reduce the heterogeneity of treatment effects, we also excluded 20 patients who underwent intra-arterial thrombolysis. In addition, 21 patients with no measurable cancellous bone (excessively narrow intercortical space of the frontal skull) were excluded. The remaining 600 cardioembolic stroke patients were included in our analyses.

This study was approved by the Institutional Review Board of our hospital and conformed to the tenets of the Declaration of Helsinki. Because this was a retrospective study, the requirement for informed consent was waived. All individual records were anonymized prior to analysis.

### 2.2. Skull HU and BMD Registry

We previously reported on the Skull HU and BMD (SHUB) registry in our hospital [11]. In addition to the previous registry (from 1 January 2010 to 31 December 2016), we further included patients (>18 years old) who had one or more procedure codes for dual-energy X-ray absorptiometry (DXA) (NMF03) and brain CT (RCG01A and B) between 1 January 2017 and 31 December 2019. According to the same protocol as before, we used the lowest T-score value for patients who underwent multiple DXA scans. When patients received multiple brain CT scans, we used the scan closest to the date of the selected DXA scan for the analysis. We excluded patients with more than three years between DXA and brain CT to reduce time interval heterogeneity. According to a study reporting a slow progression to osteoporosis in older women, we deemed our three-year time interval between the DXA and brain CT scans to be tolerable for our evaluation of the relationship between frontal skull HU and BMD [12]. In addition, we excluded patients that presented excessively narrow intercortical space of the frontal skull on the brain CT. In that way, we included 2025 patients in the updated SHUB registry.

In all registry patients, BMD was assessed in the lumbar spine (L1–L4) and femoral neck using a Discovery Wi DXA system (Hologic, Bedford, MA, USA). Each individual’s BMD was converted into a T-score based on the BMD values of a healthy young Asian reference population. We used the lower T-score between the lumbar spine and femoral neck as the T-score for the registry. Based on the World Health Organization T-score classification, we defined osteoporosis as a T-score ≤ −2.5, osteopenia as a T-score > −2.5 and ≤ −1.0, and a normal BMD as a T-score > −1.0.

This study was approved by the Institutional Review Board of our hospital. Because of its retrospective nature, the need for informed consent was waived.

### 2.3. Measuring Frontal Skull HU Values

All CT images were obtained using a Siemens CT scanner in our hospital with continuous slices, no gap, and 4.0–5.0-mm slice thickness. We previously reported detailed methods for measuring HU values on frontal cancellous bone using brain CT [11,13]. The HU values were measured at each of the four lines on the frontal cancellous bone between the right and left coronal sutures at the point at which the lateral ventricles disappear on the brain CT scan (Supplementary Figure S1). To avoid including cortical bone, we magnified all CT images for the HU value measurement. All frontal skull HU measurements were performed by two faculty neurosurgeons blinded to the clinical data of all patients.

### 2.4. Radiographic and Clinical Variables

Following the lead of a previous study, we classified all patients with HT into four categories: (1) HI1 (small petechiae without space-occupying effect), (2) HI2 (more confluent petechiae without space-occupying effect), (3) PH1 (≤30% of the infarcted area with some mild space-occupying effect), and (4) PH2 (>30% of the infarcted area with significant space-occupying effect) [2]. As described in the Introduction, we used only PH-type HT as the dependent variable in our analyses. We present examples of PH1 and PH2 HT in our study patients with cardioembolic stroke in Supplementary Figure S2. The infarction volume was measured using the ABC/2 method [14]. The diffusion-weighted image with the largest acute infarction area was selected for the volume measurement. The detailed method is shown in Supplementary Figure S3. 

Clinical data, sex, age, height, weight, initial National Institutes of Health Stroke Scale (NIHSS) score, use of tissue plasminogen activator (tPA), heart disease, previous stroke history, hypertension, diabetes, current smoking, hyperlipidemia, and prior antithrombotic use were investigated using electronic medical records. Histories of hypertension and diabetes were recorded in the electronic medical records of patients taking antihypertensive or antidiabetic medications on admission, in previous medical records indicating a history of hypertension or diabetes, or through self-reporting by the patient/guardian. Body mass index (BMI) was calculated as weight/(height)^2^ and expressed as kg/m^2^. We investigated patients’ platelet count at admission for cardioembolic stroke and defined thrombocytopenia as a platelet count <150 × 10^3^/μL.

### 2.5. Statistical Methods

Chi-square and Student’s *t* testing were used to identify differences between the PH-type HT (−) and PH-type HT (+) groups. Statistical comparisons between the three age groups were performed using one-way ANOVA. The mean skull HU value ((mean right lateral HU + mean right medial HU + mean left medial HU + mean left lateral HU)/4) was used in all analyses.

A receiver operating characteristic (ROC) curve analysis was conducted to determine the optimal cut-off values of mean skull HU for predicting osteopenia and osteoporosis in the SHUB registry patients. The optimal cut-off value was defined as the shortest distance from the upper left corner. The distance between each point on the ROC curve and the upper left corner was calculated as square root of (1−sensitivity)^2^ + (1−specificity)^2^ [15]. Mean individual skull HU values were entered as the test variable, and the individual BMD classification was used as the state variable (dependent variable) in the ROC curve analysis. When we determined the cut-off skull HU value for predicting osteopenia, we coded the normal BMD (T-score > −1.0) as 0 and the osteopenia and osteoporosis BMD (T-score ≤ −1.0) as 1, and input the state variable. In the osteoporosis model, we coded the normal and osteopenia BMD (T-score > −2.5) as 0 and osteoporosis (T-score ≤ −2.5) as 1, and input the state variable.

The cumulative hazard for PH-type HT was visualized using a Kaplan–Meier analysis classified by hypothetical BMD classification, with censoring of patients who had no PH-type HT on their last brain CT/MRI within 30 days of the day of cardioembolic stroke diagnosis. Hazard ratios (HRs) with 95% confidence intervals (CIs) were then estimated using a Cox regression analysis to identify whether the osteoporotic condition independently predicted the development of PH-type HT in patients with cardioembolic stroke. 

To balance the stroke severity between the PH-type HT (+) and PH-type HT (−) groups, additional propensity score-matched analysis was conducted using a multivariable logistic regression model. This model included the sex, age, stroke severity (NIHSS), and infarction volume. We matched the PH-type HT (+) group with the controls in a 1:2 ratio based on the greedy nearest neighbor method using R software [16].

A *p* value <0.05 was considered statistically significant. All statistical analyses were performed using R software version 3.6.3 (R Foundation for Statistical Computing, FreeSoftware Foundation, Boston, MA, USA) and SPSS for Windows, version 24.0 (IBM, Chicago, IL, USA).

## 3. Results

### 3.1. Optimal Skull HU Values Predicting Osteopenia and Osteoporosis

**Figure 1:**
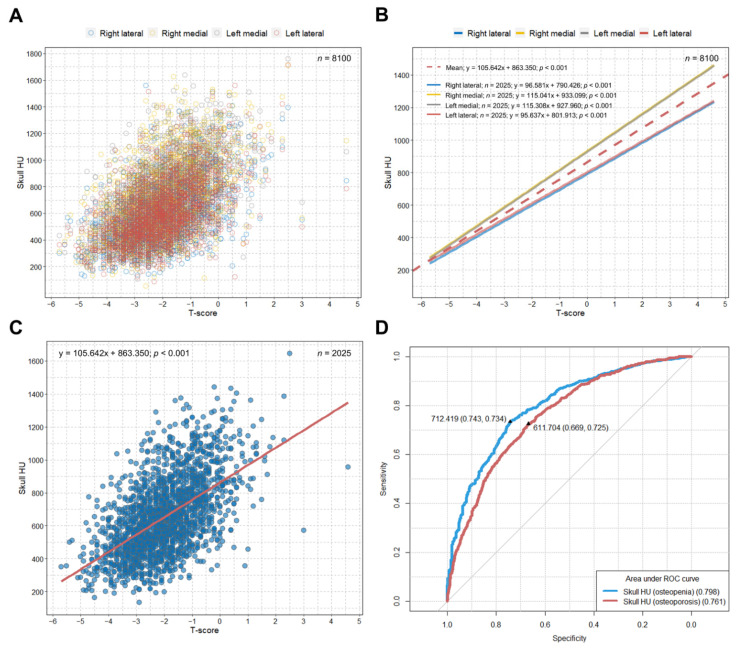
Scatterplot, linear regression line, and ROC curve from the SHUB registry. (**A**) Overall distribution of HU values at each of four lines based on T-scores; (**B**) linear association between the HU value at each of four lines and the T-score; (**C**) linear regression line showing the association between the T-score and mean skull HU; (**D**) ROC curve to identify the optimal cut-off skull HU values for predicting osteopenia and osteoporosis. ROC = receiver operating characteristic; SHUB = skull Hounsfield unit and bone mineral density; HU = Hounsfield unit.

We observed significant positive correlations between T-scores and HU values at four different sites on the frontal cancellous bone in patients from the SHUB registry (Figure 1A,B).

We observed an increase of approximately 106 mean skull HU per one T-score increase (B = 105.64; *p* < 0.001) (Figure 1C). The optimal cut-off value for mean skull HU to predict osteopenia was 712.419 (area under the curve (AUC) = 0.798; sensitivity = 73.4%; specificity = 74.3%; *p* < 0.001), and the value to predict osteoporosis was 611.704 (AUC = 0.761; sensitivity = 72.5%; specificity = 66.9%; *p* < 0.001) among the patients in the SHUB registry (Figure 1D). Based on these cut-off values, we categorized our study patients as hypothetical normal (above the cut-off HU value for osteopenia (>712.419)), osteopenia (between the cut-off HU values for osteopenia and osteoporosis (>611.704 and ≤712.419)), and osteoporosis (below the cut-off HU value for osteoporosis (≤611.704)).

### 3.2. Characteristics of Patients in the Study Cohort

Altogether, 600 consecutive patients (>18 years old) with cardioembolic stroke were enrolled over a 12-year period at our hospital. In total, 42 patients (7.0%) showed PH-type HT within 30 days of their cardioembolic stroke diagnosis (Table 1).

The median time to PH-type HT development was two days. The mean patient age was 73.6 years, and 47.7% of the patients were male. The median NIHSS at admission was five, and the mean cerebral infarction volume was 44.4 cc. A total of 252 patients (42.0%) were categorized as having hypothetical osteoporosis. Further descriptive data are shown in Table 1.

### 3.3. Skull HU Values and BMD in the Study and SHUB-Registry Cohorts

Table 2 shows detailed information about the skull HU values in both cohorts, with additional BMD information for the SHUB registry patients.

The SHUB registry had a higher proportion of women, whereas the study cohort showed an older age distribution. The overall average mean frontal skull HU value was 698.0 among the study patients and 653.0 among the SHUB registry patients. In the SHUB registry, the median time between brain CT and BMD measurement was 151 days, and 742 patients (36.6%) had osteoporosis.

### 3.4. Relationship between Age and Skull HU according to the Development of PH-Type HT 

**Figure 2:**
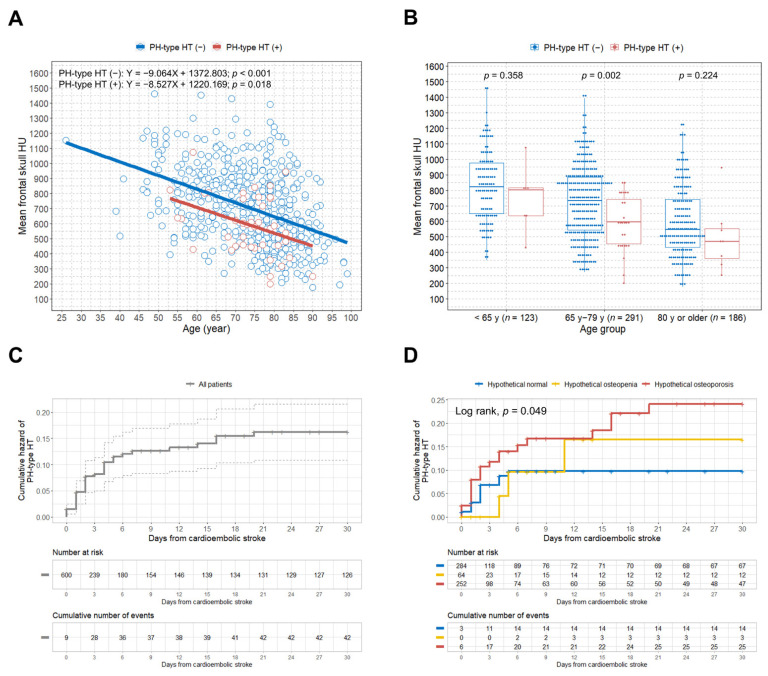
Scatterplot with linear regression lines, boxplots, and Kaplan–Meier curves in the study patients. (**A**) Scatterplot with linear regression lines showing the association between age and mean frontal skull HU values based on the presence of PH-type HT; (**B**) boxplots with dot plots of the mean frontal skull HU values classified by age group according to the presence of PH-type HT; (**C**) overall cumulative hazard of PH-type HT development; (**D**) cumulative hazard of PH-type HT development according to the hypothetical BMD groups. HU = Hounsfield unit; PH = parenchymal hematoma; HT = hemorrhagic transformation; BMD = bone mineral density.

We observed significant negative correlations between age and mean skull HU values in both the PH-type HT (−) and PH-type HT (+) groups (Figure 2A).

Overall, lower mean skull HU values were observed across the age ranges in the PH-type HT (+) group, compared with the PH-type HT (−) group. To compare the association between PH-type HT and hypothetical osteoporosis among different age groups, we classified all study participants into three age groups (<65 years, 65–79 years, ≥80 years) [17,18]. Figure 2B shows that the PH-type HT (+) group had a statistically significant lower mean skull HU value than the PH-type HT (−) group in the 65 to 79 years age group (*p* = 0.002). Data on PH-type HT and hypothetical BMD classification according to three different age groups are presented in Supplementary Table S1. The hypothetical osteoporosis group showed a significant association with PH-type HT development after cardioembolic stroke among patients aged 65–79 years (Supplementary Table S2).

### 3.5. Association between Hypothetical Osteoporosis and PH-Type HT in Cardioembolic Stroke

**Figure 3:**
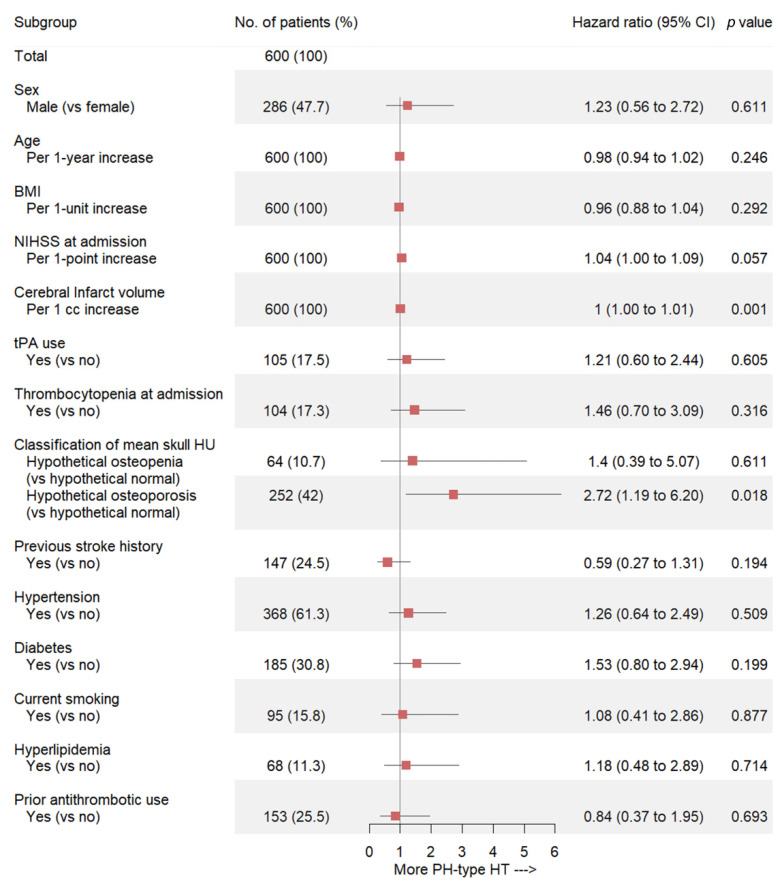
Forest plot of estimates from the multivariate Cox regression analysis to predict PH-type HT occurrence using potential predictive factors (adjusted for sex, age (continuous variable), BMI (continuous variable), NIHSS at admission (continuous variable), cerebral infarct volume (continuous variable), tPA use, thrombocytopenia at admission, classification of mean skull HU, previous stroke history, hypertension, diabetes, current smoking, hyperlipidemia, and prior antithrombotic use) in the study patients. PH = parenchymal hematoma; HT = hemorrhagic transformation; BMI = body mass index; NIHSS = National Institutes of Health Stroke Scale; tPA = tissue plasminogen activator; HU = Hounsfield unit.

Figure 2C shows the overall cumulative hazard for PH-type HT development within 30 days of cardioembolic stroke diagnosis. When we classified patients by their hypothetical BMD, the hypothetical osteoporosis group showed a significantly higher rate of PH-type HT occurrence (*p* = 0.049) (Figure 2D). The multivariate Cox regression analysis determined that infarction volume and hypothetical osteoporosis were independent predictive factors for the development of PH-type HT in patients with cardioembolic stroke (HR, 1.004; 95% CI, 1.002–1.007; *p* = 0.001; HR, 2.72; 95% CI, 1.19–6.20; *p* = 0.018, respectively) (Figure 3).

The result of our univariate Cox regression analysis is presented in Supplementary Table S3.

**Figure 4:**
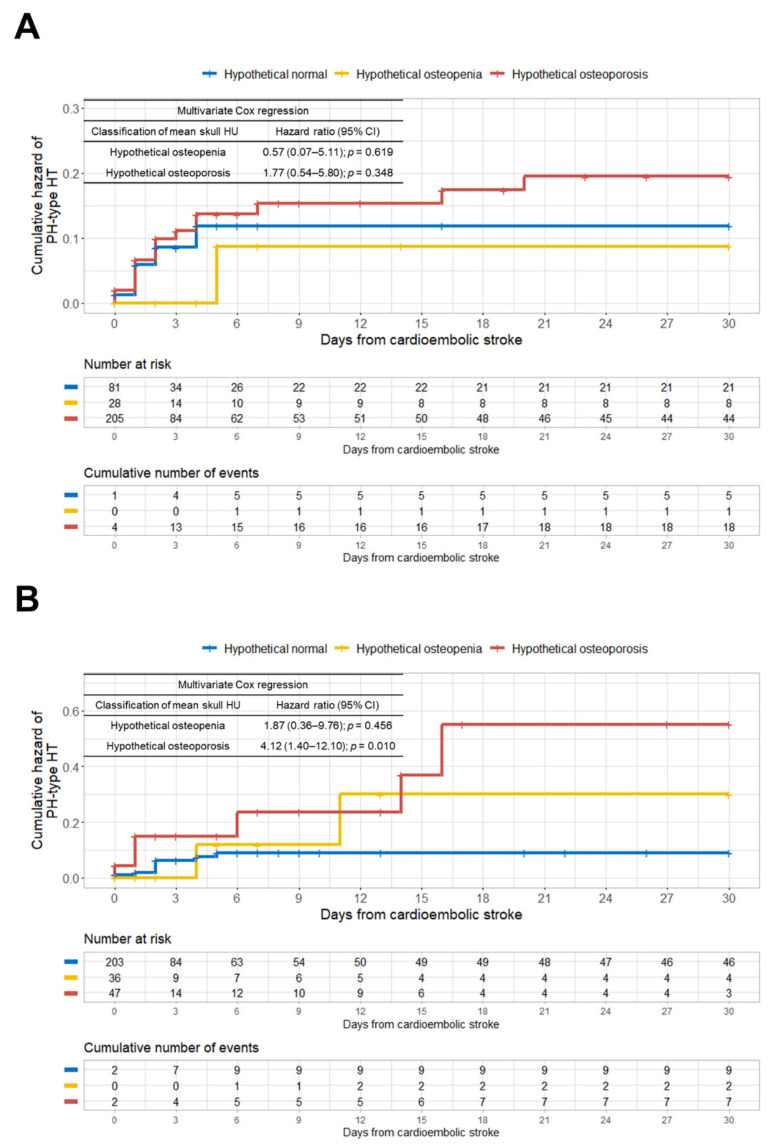
Kaplan–Meier curves showing the cumulative hazard and hazard ratio estimated by the multivariate Cox regression analysis for the development of PH-type HT in the study patients according to the hypothetical BMD groups classified by sex. (**A**) Female; (**B**) male. PH = parenchymal hematoma; HT = hemorrhagic transformation; BMD = bone mineral density.

When we classified the patients by sex, we found a significant discrepancy in the prevalence of hypothetical osteoporosis between men and women (Figure 4).

The rate of hypothetical osteoporosis in women (65.3% (205/314)) was about four times higher than in men (16.4% (47/286)). Supplementary Figure S4 shows the very different correlations between age and mean skull HU values between women and men. In contrast to the women, we found no significant linear association between age and mean skull HU in males. Figure 4A showed that the female group had about 2.5-fold more hypothetical osteoporosis patients (*n* = 205) than hypothetical normal patients (*n* = 81). We found no significant association between hypothetical osteoporosis and the development of PH-type HT in the multivariate Cox analysis in the female group. However, in the male group, hypothetical osteoporosis was an independent predictor for the development of PH-type HT after cardioembolic stroke (HR, 4.12; 95% CI, 1.40–12.10; *p* = 0.010) (Figure 4B). We present the detailed results of our uni- and multivariate Cox regression analyses of the female and male groups in Supplementary Tables S4 and S5, respectively. To control for the effect of stroke severity between the PH-type HT (+) and PH-type HT (−) groups, we performed an additional propensity score matching analysis based on four covariates, the sex, age, stroke severity (NIHSS), and infarction volume (Table 3).

We observed that hypothetical osteoporosis remained an independent predictive factor for the development of PH-type HT after cardioembolic stroke in the propensity score-matched male patients (HR, 3.29; 95% CI, 1.18–9.17; *p* = 0.023) (Table 4).

## 4. Discussion

We found that hypothetical low BMD, as determined by skull HU values from brain CT scans, is associated with an increased risk of PH-type HT in patients with cardioembolic stroke. In men, the hypothetical osteoporosis group showed an approximately four-fold higher risk of PH-type HT after cardioembolic stroke than the hypothetical normal group after adjusting for other predictive factors, including age. It has been reported that women ≥ 50 years of age have a four times higher rate of osteoporosis, compared with men [19]. We also found that women had about a four-fold higher rate of possible osteoporosis than men. However, unlike males, females showed no significant relationship between hypothetical osteoporosis and PH-type HT occurrence after a cardioembolic stroke. Previous studies reported that a 1:4 case–control ratio provides suitable statistical power [20,21]. Therefore, the case (hypothetical osteoporosis, *n* = 47): control (hypothetical normal, *n* = 203) ratio of 1:4.3 in our group of male patients has higher statistical power than our group of female patients, which contained more cases (hypothetical osteoporosis, *n* = 205) than controls (hypothetical normal, *n* = 81). In addition, when we controlled for the effect of stroke severity between the PH-type HT (−) and PH-type HT (+) groups using the propensity score matching analysis, the hypothetical osteoporosis group still showed a statistically significant association with PH-type HT development after cardioembolic stroke in males. To the best of our knowledge, this is the first study to suggest an association between possible osteoporosis and the development of PH-type HT in cardioembolic stroke. 

Both HU in a specific anatomical area on a CT image and BMD T-scores are absolute values [22,23]. Therefore, we suggest that the relationship between those absolute values might not be affected by the heterogeneous patient characteristics between our study cohort and the SHUB registry. Patient characteristics can affect bone quality, however they might not affect the simple association between the absolute values of HU and BMD [11]. Furthermore, the relationship between those absolute values should be linear, even if the cohort is highly heterogeneous. Similar to our study, Pickhardt et al. demonstrated an optimal HU threshold for predicting osteoporosis among heterogeneous patients using lumbar spine HU values from abdominal CT images obtained for other reasons [24]. Other studies have also reported that cancellous bone HU values from CT scans at specific anatomic regions show a strong association with T-scores and can be useful for detecting osteoporosis [25,26]. We previously demonstrated that the HU value of cancellous bone at the frontal skull can predict osteoporotic conditions [11]. In general, ROC curves with an AUC >0.75 may be clinically meaningful [27]. Although there was considerable scattering between T-scores and skull HU values, we observed that the ROC curve analysis of the mean skull HU with a large sample size (*n* = 2025) showed AUCs >0.75 for the prediction of both osteopenia and osteoporosis.

Because osteoporosis is a systemic disease that influences systemic bone mass and microarchitecture throughout the body, it is reasonable to expect that osteoporotic conditions could influence the cancellous bone of the frontal skull [28]. Although osteoporosis is multifactorial, genetic factors play an important role in determining bone density and the pathogenesis of osteoporosis [29,30]. Osteoporosis is also known to be strongly related to the genetic components of type I collagen (*COL1A1 and COL1A2*) [6]. It is well known that type I collagen is a major bone component, and mutation of its genes can cause osteoporosis [6,31]. Type I collagen is found in the extracellular matrix (ECM) of all three tunicae of the blood vessel, especially around the smooth muscle cells of the media, where it provides the necessary mechanical strength and contractility [9]. Therefore, we hypothesized that systemic osteoporosis might negatively influence the structural integrity of the ECM in blood vessels, especially around the smooth muscle cells of the media. The loss and degenerative changes of smooth muscle cells in blood vessels is a major histopathological feature of an intracranial aneurysm, and it is well known that intracranial aneurysms can easily rupture and produce subarachnoid hemorrhages [8]. Supporting our hypothesis, osteogenesis imperfecta caused by mutations in type I collagen genes (*COL1A1/COL1A2*) is associated with hemorrhagic diathesis, and is also hypothesized to be associated with vascular fragility [32,33]. In addition, previous studies reported abnormalities in the cerebral arterial system in osteogenesis imperfecta [34,35,36]. We believe that these findings were explained as vasculopathic changes, secondary to vascular fragility caused by collagen type I abnormalities in osteogenesis imperfecta. In addition, HT, including PH-type HT, is more prevalent in cardioembolic stroke than in other types of ischemic stroke [37]. Therefore, weakened ECM around smooth muscle cells in blood vessels caused by osteoporotic conditions might make those vessels more vulnerable to rupture and hemorrhage after a cardioembolic stroke. Based on that concept, our results can be explained by the following hypothetical mechanism: when reperfusion occurs, blood perfusion can again put pressure on the vessel wall, and the weakened ECM around the vascular smooth muscle cells caused by osteoporotic conditions could be more likely to rupture than the vessels in patients with stronger ECM around the vascular smooth muscle cells, producing larger hematomas, such as PH-type HT, after cardioembolic stroke.

Despite these explanations, there is a discrepancy between a higher prevalence of osteoporosis in females and no significant difference in PH-type HT occurrence between males and females. However, males with ICH showed a higher risk of hematoma expansion and both early and late mortality compared to females [38]. In addition, males had more osteoporosis-related complications and a higher mortality rate than females with osteoporotic fractures [19,39]. Moreover, estrogen plays a role in maintaining adequate cerebral perfusion which may suggest sex differences in hemorrhage expansion and mortality [40]. As an antioxidant, estrogen acts as a neuroprotectant that reduces free radicals and decreases BBB permeability and edema [41,42]. Given the age distribution of our female patients, most were likely postmenopausal. Although our study lacked information on exposure to hormone replacement therapy, we believe that there may have been some protective effect of estrogen on PH-type HT development after cardioembolic stroke in female patients. However, our findings need to be confirmed by future studies.

Another possible predictive factor for PH-type HT after a cardioembolic stroke in our results was a larger cerebral infarction volume, which is consistent with previous reports about ischemic stroke [1,37]. In addition, massive cerebral infarction and enhanced permeability of the vascular wall caused by ischemia and hypoxia may also cause PH-type HT development after cardioembolic stroke, independent of the osteoporotic condition [1].

Our study has some limitations. First, its retrospective nature carries inherent limitations. Second, we did not have actual T-scores for the study patients because ischemic stroke patients rarely receive DXA scans. However, we showed that frontal cancellous bone HU values have relatively high specificity and sensitivity in predicting actual T-scores. Third, HU measurement errors might have occurred, especially in patients with a narrow intercortical space in the frontal bone. However, we magnified all the brain CT scans for HU measurement and initially excluded patients with no measurable intercortical space, as described in the Methods. In addition, using mean HU values from four areas of the frontal skull could reduce that bias. Finally, in our study, the relatively low outcome event rates may affect the results of the statistical analysis and have limited the predictive ability.

## 5. Conclusions

In conclusion, our results suggest an association between possible osteoporosis and the development of PH-type HT in patients with cardioembolic stroke. However, the association between possible osteoporosis and the development of PH-type HT after cardioembolic stroke was significant in males, but not females. Our findings can help to predict the development of PH-type HT during the clinical course of cardioembolic stroke by providing a convenient method for measuring the HU value of the frontal skull on brain CT. However, further studies including other types of ischemic stroke are required to confirm these initial findings. We expect our findings to help improve understanding about the underlying mechanism of the relationship between HT in acute ischemic stroke and BMD.

## Figures and Tables

**Table 1:** Characteristics of patients with cardioembolic cerebral infarction classified according to the presence of HT.

Characteristics	PH-Type HT (−)	PH-Type HT(+)	Total	*p*
Number (%)	558 (93.0)	42 (7.0)	600 (100)	
Time to PH-type HT development, median (IQR), day	N/A	2.0 (1.0–4.0)	N/A	
Sex, male, *n* (%)	268 (48.0)	18 (42.9)	286 (47.7)	0.518
Age, mean ± SD, y	73.6 ± 11.1	73.6 ± 8.4	73.6 ± 11.0	0.980
BMI, mean ± SD, kg/m^2^	23.5 ± 4.0	23.0 ± 3.4	23.4 ± 4.0	0.449
Height, mean ± SD, cm	160.5 ± 9.1	159.2 ± 7.1	160.4 ± 9.0	0.344
Weight, mean ± SD, kg	60.9 ± 12.9	58.4 ± 9.8	60.7 ± 12.7	0.220
NIHSS at admission, mean ± SD	7.5 ± 7.0	12.3 ± 6.3	7.8 ± 7.1	<0.001
NIHSS at admission, median (IQR)	5.0 (2.0–12.0)	12.5 (7.0–17.0)	5.0 (2.0–12.0)	<0.001
Cerebral infarct volume, mean ± SD, cc	39.5 ± 76.3	108.8 ± 103.5	44.4 ± 80.3	<0.001
tPA use, *n* (%)	93 (16.7)	12 (28.6)	105 (17.5)	0.050
Platelet count at admission, mean ± SD, × 10^3^/μL	203.1 ± 66.0	199.6 ± 64.0	202.8 ± 65.8	0.744
Mean frontal skull HU, mean ± SD	705.9 ± 247.6	592.4 ± 197.6	698.0 ± 246.0	0.004
Classification of mean skull HU, *n* (%)				0.058
Hypothetical normal (>712.4)	270 (48.4)	14 (33.3)	284 (47.3)	
Hypothetical osteopenia (>611.7 and ≤712.4)	61 (10.9)	3 (7.1)	64 (10.7)	
Hypothetical osteoporosis (≤611.7)	227 (40.7)	25 (59.5)	252 (42.0)	
Heart disease, *n* (%)				0.375
Arrhythmia	399 (71.5)	36 (85.7)	435 (72.5)	
CHF, cardiomyopathy, valvular heart disease	90 (16.1)	3 (7.1)	93 (15.5)	
Coronary artery disease	34 (6.1)	1 (2.4)	35 (5.8)	
Previous MI	34 (6.1)	2 (4.8)	36 (6.0)	
Cardiac myxoma	1 (0.2)	0	1 (0.2)	
Past medical history, *n* (%)				
Previous stroke history	139 (24.9)	8 (19.0)	147 (24.5)	0.394
Hypertension	340 (60.9)	28 (66.7)	368 (61.3)	0.462
Diabetes	169 (30.3)	16 (38.1)	185 (30.8)	0.291
Current smoking	88 (15.8)	7 (16.7)	95 (15.8)	0.878
Hyperlipidemia	62 (11.1)	6 (14.3)	68 (11.3)	0.531
Prior antithrombotic use	146 (26.2)	7 (16.7)	153 (25.5)	0.173

PH, parenchymal hematoma; HT, hemorrhagic transformation; SD, standard deviation; BMI, body mass index; NIHSS, National Institutes of Health Stroke Scale; IQR, interquartile range; tPA, tissue plasminogen activator; HU, Hounsfield unit; CHF, congestive heart failure; MI, myocardial infarction.

**Table 2:** Descriptive information about skull HU values and BMD in the study and SHUB registry cohorts.

Variables	Study Cohort	SHUB Registry
Number	600	2025
Sex		
Female, *n* (%)	314 (52.3)	1704 (84.1)
Age, median (IQR), y	75.5 (67.0–81.0)	69.0 (59.0–77.0)
Age, mean ± SD, y	73.6 ± 11.0	67.9 ± 11.9
Overall mean skull HU value, median (IQR)	681.0 (507.6–872.6)	625.7 (483.8–791.5)
Overall mean skull HU value, mean ± SD	698.0 ± 246.0	653.0 ± 229.9
Mean HU value at each of four sites in the frontal skull, mean ± SD		
Right lateral	642.9 ± 224.5	598.1 ± 220.4
Right medial	763.3 ± 294.0	704.1 ± 263.6
Left medial	750.0 ± 289.1	698.4 ± 262.4
Left lateral	635.8 ± 230.6	611.5 ± 224.8
Average, medial	756.7 ± 286.9	701.2 ± 257.9
Average, lateral	639.3 ± 221.0	604.8 ± 216.5
Time interval between brain CT and BMD, median (IQR), days	N/A	151.0 (9.0–487.0)
T-score, mean ± SD	N/A	−1.99 ± 1.22
Lumbar spine	N/A	−1.65 ± 1.43
Femur neck	N/A	−1.40 ± 1.20
BMD categories, *n* (%)		
Normal (T-score > −1.0)	N/A	381 (18.8)
Osteopenia (T-score > −2.5 and ≤ 1.0)	N/A	902 (44.5)
Osteoporosis (T-score ≤ −2.5)	N/A	742 (36.6)

HU, Hounsfield unit; BMD, bone mineral density; SHUB, skull Hounsfield unit and bone mineral density; IQR, interquartile range; SD, standard deviation; CT, computed tomography; N/A, not available.

**Table 3:** Characteristics of patients with cardioembolic cerebral infarction classified according to the presence of PH-type HT before and after propensity score matching based on the sex, age, stroke severity (NIHSS), and infarction volume.

Characteristics	Before Propensity Score Matching			After Propensity Score Matching		
	PH-Type HT (−) (*n* = 558)	PH-Type HT (+) (*n* = 42)	*p*	PH-Type HT (−) (*n* = 84)	PH-Type HT (+) (*n* = 42)	*p*
Sex, male, *n* (%)	268 (48.0)	18 (42.9)	0.518	37 (44.0)	18 (42.9)	0.899
Age, mean ± SD, y	73.6 ± 11.1	73.6 ± 8.4	0.980	75.1 ± 10.7	73.6 ± 8.4	0.434
NIHSS at admission, mean ± SD	7.5 ± 7.0	12.3 ± 6.3	<0.001	13.7 ± 6.3	12.3 ± 6.3	0.226
NIHSS at admission, median (IQR)	5.0 (2.0–12.0)	12.5 (7.0–17.0)	<0.001	13.5 (10.0–18.0)	12.5 (7.0–17.0)	0.226
Cerebral infarct volume, mean ± SD, cc	39.5 ± 76.3	108.8 ± 103.5	<0.001	92.0 ± 103.3	108.8 ± 103.5	0.390
Cerebral infarct volume, median (IQR)	6.7 (0.4–40.6)	88.5 (17.4–166.3)	<0.001	52.6 (11.0–133.4)	88.5 (17.4–166.3)	0.390
Classification of mean skull HU, *n* (%)			0.058			0.376
Hypothetical normal (>712.4)	270 (48.4)	14 (33.3)		38 (45.2)	14 (33.3)	
Hypothetical osteopenia (>611.7 and ≤712.4)	61 (10.9)	3 (7.1)		7 (8.3)	3 (7.1)	
Hypothetical osteoporosis (≤611.7)	227 (40.7)	25 (59.5)		39 (46.4)	25 (59.5)	

PH, parenchymal hematoma; HT, hemorrhagic transformation; NIHSS, National Institutes of Health Stroke Scale; SD, standard deviation; IQR, interquartile range; HU, Hounsfield unit.

**Table 4:** Multivariate Cox regression analyses of PH-type HT development after cardioembolic stroke classified by sex in propensity score matched patients (based on the sex, age, stroke severity (NIHSS), and infarction volume).

	Total (*n* = 126)		Female (*n* = 71)		Male (*n* = 55)	
Variable	HR (95% CI)	*p*	HR (95% CI)	*p*	HR (95% CI)	*p*
Sex						
Male	1.21 (0.57–2.56)	0.618	N/A		N/A	
Female	Reference		N/A		N/A	
Age (per 1–year increase)	0.98 (0.95–1.02)	0.296	0.98 (0.94–1.03)	0.493	0.99 (0.94–1.04)	0.663
NIHSS at admission (per 1–point increase)	0.96 (0.91–1.02)	0.189	0.89 (0.81–0.98)	0.015	1.02 (0.95–1.09)	0.616
Cerebral infarct volume (per 1 cc increase)	1.00 (1.00–1.00)	0.351	1.00 (1.00–1.01)	0.091	1.00 (0.99–1.00)	0.678
Classification of mean skull HU, *n* (%)						
Hypothetical normal (>712.4)	Reference		Reference		Reference	
Hypothetical osteopenia (>611.7 and ≤712.4)	1.29 (0.36–4.55)	0.697	2.12 (0.23–19.94)	0.511	1.23 (0.26–5.92)	0.795
Hypothetical osteoporosis (≤611.7)	2.24 (0.99–5.09)	0.054	1.62 (0.49–5.33)	0.429	3.29 (1.18–9.17)	0.023

PH, parenchymal hematoma; HT, hemorrhagic transformation; HR, hazard ratio; CI, confidence interval; N/A, not available; NIHSS, National Institutes of Health Stroke Scale; HU = Hounsfield unit.

## Data Availability

The data presented in this study are available on reasonable request from the corresponding author.

## Supplementary Materials

The following are available online at https://www.mdpi.com/article/10.3390/jcm10112526/s1, Figure S1: Measurement of HU values at each of the four lines on the frontal cancellous bone, Figure S2: Examples of PH type 1 and 2 HT in study patients with cardioembolic stroke, Figure S3: Measurement of the cerebral infarction volume, Figure S4. Scatterplots with linear regression lines, Table S1: Characteristics of the study individuals focusing on the PH-type HT and hypothetical BMD classification according to three different age groups, Table S2: Univariate Cox regression analyses of PH-type HT development after cardioembolic stroke according to hypothetical BMD classification based on different age groups, Table S3: Univariate Cox regression analyses of potential predictive factors of PH-type HT in patients with cardioembolic stroke, Table S4: Uni- and multivariate Cox regression analyses of potential predictive factors for PH-type HT among female patients with cardioembolic stroke, Table S5: Uni- and multivariate Cox regression analyses of potential predictive factors for PH-type HT among male patients with cardioembolic stroke.
