# Supplementary material for: Effect of Possible Osteoporosis on Parenchymal-Type Hemorrhagic Transformation in Patients with Cardioembolic Stroke
Source: J Clin Med. 2021 Jun 7;10(11):2526. doi: 10.3390/jcm10112526 (PMC8201205; doi:10.3390/jcm10112526)
Supplement: Supplementary file 1 [file jcm-10-02526-s001.zip › jcm-1220167-supplementary.pdf]

Supplementary Materials

Supplemental Table S1. Characteristics of the study individuals focusing on the PH-type HT and hypothetical BMD classification according to three different age groups

Characteristics	<65 years	65–79 years	≥80 years	Total	p
Number	123	291	186	600	
PH-type HT (+), n (%)	7 (5.7)	27 (9.3)	8 (4.3)	42 (7.0)	0.094
Classification of mean skull HU, n (%)					<0.001
Hypothetical normal (>712.4)	80 (65.0)	151 (51.9)	53 (28.5)	284 (47.3)	
Hypothetical osteopenia (>611.7 and ≤712.4)	19 (15.4)	26 (8.9)	19 (10.2)	64 (10.7)	
Hypothetical osteoporosis (≤611.7)	24 (19.5)	114 (39.2)	114 (61.3)	252 (42.0)	

PH, parenchymal hematoma; HT, hemorrhagic transformation; BMD, bone mineral density; HU, Hounsfield unit

Supplemental Table S2. Univariate Cox regression analyses of PH-type HT development after cardioembolic stroke according to hypothetical BMD classification based on different age groups

	<65 years (n=123)		65–79 years (n=291)		≥80 years (n=186)	
Variable	HR (95% CI)	p	HR (95% CI)	p	HR (95% CI)	p
Classification of mean skull HU, n (%)						
Hypothetical normal (>712.4)	Reference		Reference		Reference	
Hypothetical osteopenia (>611.7 and ≤712.4)	3.59 (0.65–19.97)	0.144	0.62 (0.08–4.91)	0.652	0.00 (0.00–0.00)	0.986
Hypothetical osteoporosis (≤611.7)	2.18 (0.23–20.93)	0.499	2.28 (1.02–5.13)	0.045	3.09 (0.38–25.10)	0.292

PH, parenchymal hematoma; HT, hemorrhagic transformation; BMD, bone mineral density; HR, hazard ratio; CI, confidence interval; HU=Hounsfield unit

Supplemental Table S3. Univariate Cox regression analyses of potential predictive factors of PH-type HT in patients with cardioembolic stroke

Variable	HR (95% CI)	p
Sex		
Male	1.13 (0.61–2.08)	0.700
Female	Reference	
Age (per 1-year increase)	1.00 (0.97–1.03)	0.996
BMI (per 1-unit increase)	0.97 (0.90–1.05)	0.462
NIHSS at admission (per 1-point increase)	1.07 (1.03–1.10)	<0.001
Cerebral infarct volume (per 1 cc increase)	1.01 (1.00–1.01)	<0.001
tPA use		
No	Reference	
Yes	1.55 (0.80–3.04)	0.198
Thrombocytopenia at admission		
No	Reference	
Yes	1.81 (0.89–3.69)	0.102
Classification of mean skull HU		
Hypothetical normal	Reference	
Hypothetical osteopenia	1.04 (0.30–3.64)	0.946
Hypothetical osteoporosis	2.13 (1.11–4.09)	0.024
Previous stroke history		
No	Reference	
Yes	0.54 (0.25–1.17)	0.116
Hypertension		
No	Reference	
Yes	1.20 (0.63–2.28)	0.575
Diabetes		
No	Reference	
Yes	1.35 (0.72–2.51)	0.352
Current smoking		
No	Reference	
Yes	1.13 (0.50–2.54)	0.771
Hyperlipidemia		
No	Reference	

Yes	1.25 (0.53–2.96)	0.619
Prior antithrombotic use		
No	Reference	
Yes	0.65 (0.29–1.46)	0.295

PH, parenchymal hematoma; HT, hemorrhagic transformation; HR, hazard ratio; CI, confidence interval; BMI, body mass index; NIHSS, National Institutes of Health Stroke Scale; tPA, tissue plasminogen activator; HU, Hounsfield unit

Supplemental Table S4. Uni- and multivariate Cox regression analyses of potential predictive factors for PH-type HT among female patients with cardioembolic stroke

	Univariate		Multivariate	
Variable	HR (95% CI)	p	HR (95% CI)	p
Age (per 1-year increase)	0.99 (0.95–1.03)	0.498	0.96 (0.91–1.01)	0.100
BMI (per 1-unit increase)	0.98 (0.89–1.08)	0.735	0.97 (0.87–1.09)	0.606
NIHSS at admission (per 1-point increase)	1.05 (1.00–1.11)	0.052	1.03 (0.97–1.10)	0.302
Cerebral infarct volume (per 1 cc increase)	1.01 (1.00–1.01)	<0.001	1.00 (1.00–1.01)	0.017
tPA use				
No	Reference		Reference	
Yes	1.15 (0.43–3.09)	0.778	1.00 (0.35–2.82)	0.993
Thrombocytopenia at admission				
No	Reference		Reference	
Yes	2.75 (1.09–6.95)	0.033	1.66 (0.59–4.69)	0.343
Classification of mean skull HU				
Hypothetical normal	Reference		Reference	
Hypothetical osteopenia	0.53 (0.06–4.53)	0.562	0.57 (0.07–5.11)	0.619
Hypothetical osteoporosis	1.45 (0.54–3.92)	0.459	1.77 (0.54–5.80)	0.348
Previous stroke history				
No	Reference		Reference	
Yes	0.63 (0.23–1.70)	0.359	0.54 (0.18–1.60)	0.266
Hypertension				
No	Reference		Reference	
Yes	1.50 (0.59–3.77)	0.393	1.53 (0.59–3.99)	0.380
Diabetes				
No	Reference		Reference	
Yes	1.68 (0.75–3.76)	0.204	1.73 (0.74–4.03)	0.206

Current smoking				
No	Reference		Reference	
Yes	0.05 (0.0–1299.6)	0.559	0.0 (0.0–0.0)	0.976
Hyperlipidemia				
No	Reference		Reference	
Yes	1.59 (0.54–4.65)	0.398	1.42 (0.46–4.34)	0.540
Prior antithrombotic use				
No	Reference		Reference	
Yes	0.95 (0.38–2.40)	0.916	1.37 (0.52–3.66)	0.525

PH, parenchymal hematoma; HT, hemorrhagic transformation; HR, hazard ratio; CI, confidence interval; BMI, body mass index; NIHSS, National Institutes of Health Stroke Scale; tPA, tissue plasminogen activator; HU, Hounsfield unit

Supplemental Table S5. Uni- and multivariate Cox regression analyses of potential predictive factors for PH-type HT among male patients with cardioembolic stroke

	Univariate		Multivariate	
Variable	HR (95% CI)	p	HR (95% CI)	p
Age (per 1-year increase)	1.01 (0.97–1.06)	0.664	1.01 (0.96–1.07)	0.664
BMI (per 1-unit increase)	0.96 (0.84–1.09)	0.511	0.92 (0.79–1.07)	0.277
NIHSS at admission (per 1-point increase)	1.08 (1.03–1.13)	0.002	1.07 (1.00–1.16)	0.056
Cerebral infarct volume (per 1 cc increase)	1.01 (1.00–1.01)	0.001	1.00 (1.00–1.01)	0.126
tPA use				
No	Reference		Reference	
Yes	2.17 (0.84–5.60)	0.110	1.24 (0.43–3.57)	0.695
Thrombocytopenia at admission				
No	Reference		Reference	
Yes	1.18 (0.39–3.58)	0.775	1.25 (0.39–4.00)	0.708
Classification of mean skull HU				
Hypothetical normal	Reference		Reference	
Hypothetical osteopenia	1.71 (0.37–7.97)	0.492	1.87 (0.36–9.76)	0.456
Hypothetical osteoporosis	4.27 (1.59–11.49)	0.004	4.12 (1.40–12.10)	0.010
Previous stroke history				
No	Reference		Reference	
Yes	0.43 (0.13–1.51)	0.188	0.68 (0.18–2.67)	0.584
Hypertension				
No	Reference		Reference	
Yes	0.92 (0.37–2.34)	0.868	0.88 (0.31–2.49)	0.805
Diabetes				
No	Reference		Reference	
Yes	0.96 (0.34–2.69)	0.938	1.05 (0.35–3.16)	0.925

Current smoking				
No	Reference		Reference	
Yes	1.57 (0.61–4.05)	0.353	1.94 (0.59–6.38)	0.275
Hyperlipidemia				
No	Reference		Reference	
Yes	0.86 (0.20–3.75)	0.843	1.20 (0.23–6.30)	0.833
Prior antithrombotic use				
No	Reference		Reference	
Yes	0.22 (0.03–1.65)	0.141	0.21 (0.02–1.89)	0.164

PH, parenchymal hematoma; HT, hemorrhagic transformation; HR, hazard ratio; CI, confidence interval; BMI, body mass index; NIHSS, National Institutes of Health Stroke Scale; tPA, tissue plasminogen activator; HU, Hounsfield unit

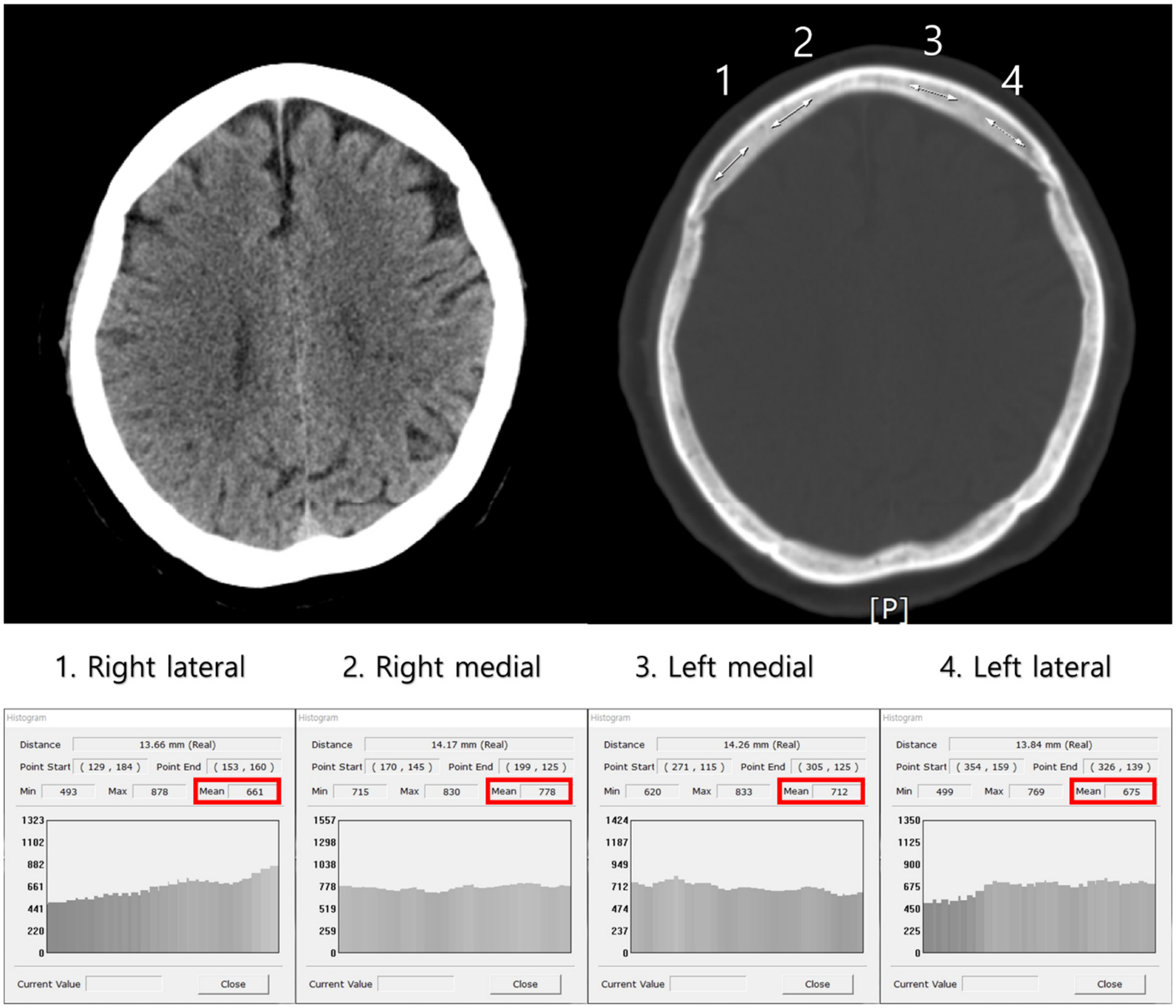

Supplemental Figure S1. Measurement of HU values at each of the four lines on the frontal cancellous bone. The mean HU value on each of the four lines was recorded for the analysis. HU=Hounsfield unit.

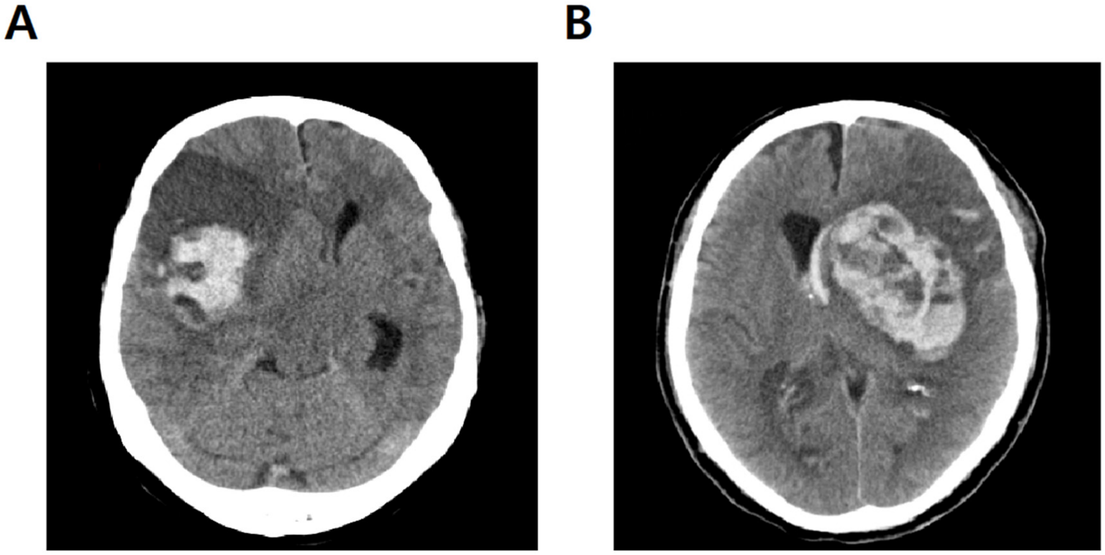

Supplemental Figure S2. Examples of PH type 1 and 2 HT in study patients with cardioembolic stroke. (A) PH1; (B) PH2. PH=parenchymal hematoma, HT=hemorrhagic transformation.

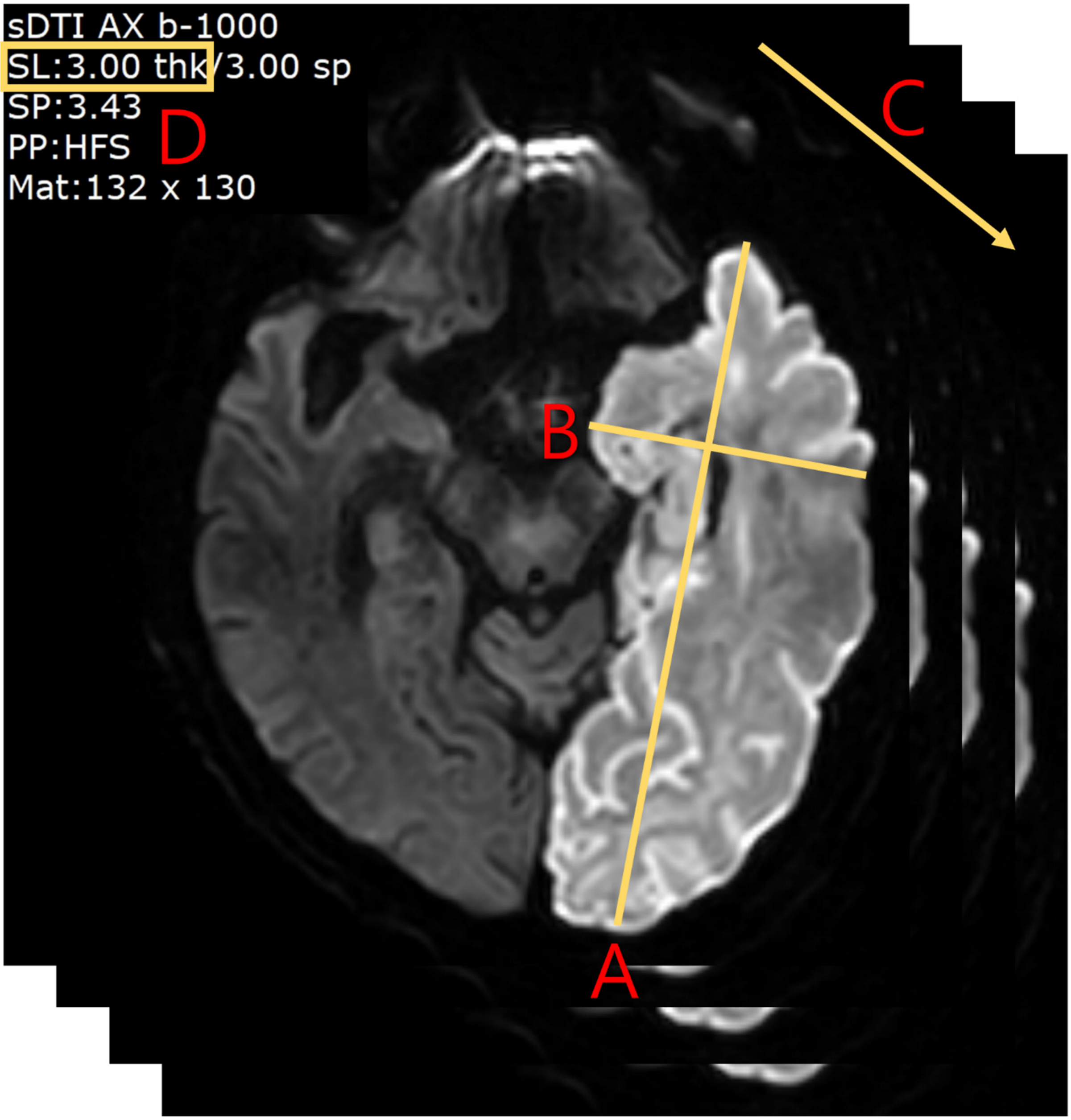

$$\text{Infarction Volume} = \frac{A \times B \times C \times D}{2}$$

Supplemental Figure S3. Measurement of the cerebral infarction volume. (A) The maximum diameter of the infarction; (B) largest diameter perpendicular to A; (C) number of images with infarction; (D) slice thickness.

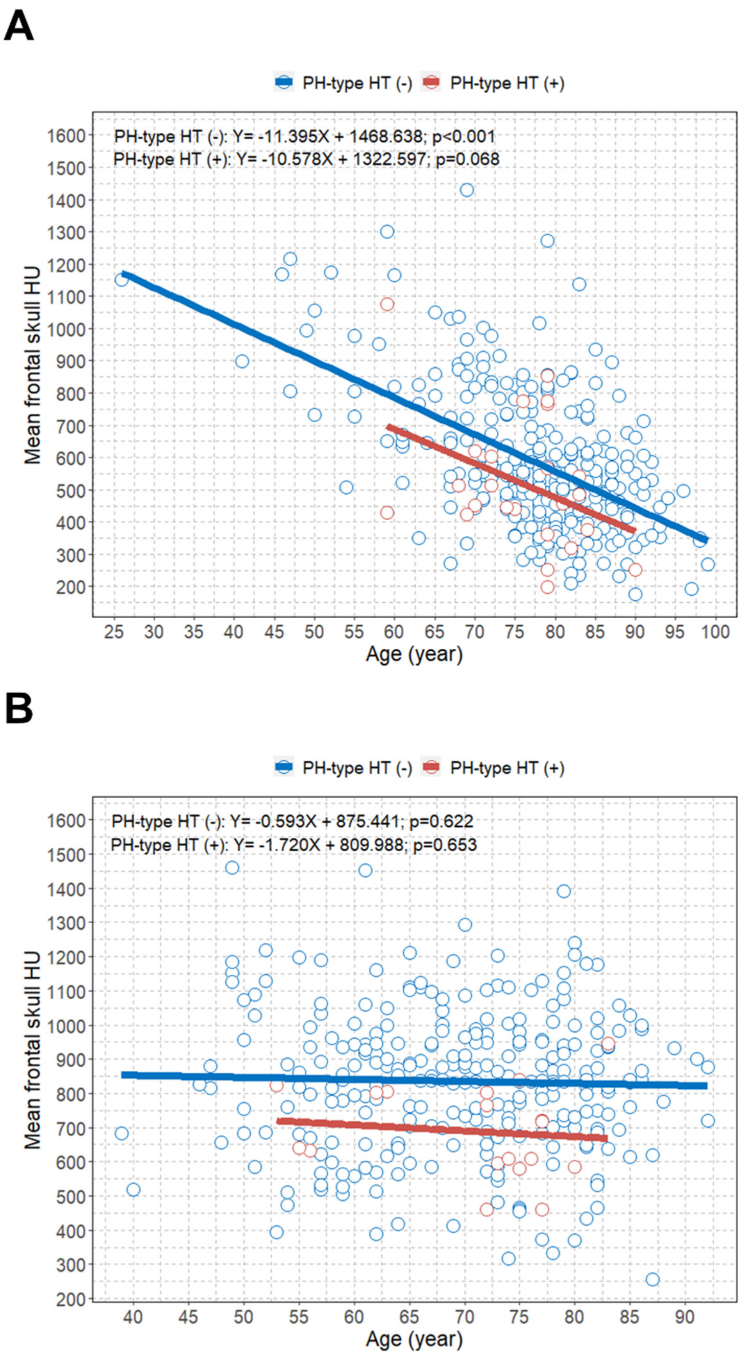

Supplemental Figure S4. Scatterplots with linear regression lines. (A) Scatterplot with the linear regression line showing the association between age and mean frontal skull HU values based on the presence of PH-type HT in women; (B) scatterplot with the linear

regression line showing the association between age and mean frontal skull HU values based on the presence of PH-type HT in males. PH=parenchymal hematoma, HT=hemorrhagic transformation, HU=Hounsfield unit.
